# Unilateral retinoblastoma; natural history and an age-based protocol in 248 patients

**DOI:** 10.1038/s41433-020-01275-2

**Published:** 2020-11-13

**Authors:** Hossam El Zomor, Radwa Nour, Anas Saad, Hala Taha, Abdallah E. Shelil, Adel Aleieldin, M. Saad Zaghloul, Ahmad S. Alfaar

**Affiliations:** 1 Egyptian National Cancer Institute, Cairo, Egypt; 2 Children’s Cancer Hospital Egypt, Cairo, 57357 Egypt; 3 London School of Hygiene and Tropical Medicine, London, UK; 4 Cleveland Clinic Foundation, Cleveland, OH USA; 5 Faculty of Medicine, Ain Shams University, Cairo, Egypt; 6 Faculty of Medicine, Al-Azhar University, Cairo, Egypt; 7 Research Institute of Ophthalmology, Giza, Egypt; 8 Experimental Ophthalmology, Department of Ophthalmology, Charité—Universitätsmedizin Berlin, Campus Virchow-Klinikum, Berlin, Germany; 9 Ophthalmology Department, Faculty of Medicine, Leipzig University, Leipzig, Germany

**Keywords:** Paediatrics, Retinal diseases

## Abstract

**Objectives:**

We aimed to study the clinical state and prognosis of patients with unilateral retinoblastoma who were being treated at a paediatric comprehensive cancer centre in a limited-resource country, to assess the different phases of treatment and the success of different, more complex real-life models.

**Subjects:**

In this retrospective study, we created a snapshot of our retinoblastoma database for the period between 2007 and 2015. Patients whose data were included in the study were followed up until 2016. Out of a total of 744 screened patients, we included data of 248 patients who had been diagnosed with unilateral retinoblastoma.

**Results:**

As classified as per the International Retinoblastoma Classification, 1 patient presented with group A, 21 with group B, 39 with group C, 104 with group D and 83 with group E retinoblastoma. Chemotherapy was the initial line of treatment in 115 patients and enucleation in 133 others. Later, 141 patients (56.9%) required further management. Patients had a mean ocular survival time of 20.8 months. Nine patients developed extraocular disease at a later stage of management: five after upfront enucleation and four after neoadjuvant chemotherapy. Mean overall survival time stood at 90.2 months. Four and three deaths were recorded in groups D and E, respectively. A single patient died in the initial chemotherapy arm, while six passed away in the initial enucleation arm.

**Conclusion:**

Our study highlights the importance of initial chemotherapy and close follow-up after enucleation of classes D and E affected eyes even in absence of germline mutations.

## Introduction

Treatment of retinoblastoma has become a success a story in developed countries with survival rates reaching 100% in this most common childhood ocular malignancy. In developing countries, unfortunately, survival rates are significantly lower [1]. Although childhood retinoblastoma is a rarity compared with other (and adult ocular) malignancies, it represents a health problem in developing countries. This is attributable to the wide-base population pyramid, imposing a heavy burden on ophthalmic oncology teams in these countries.

Retinoblastoma is the first cancer to be described using a mathematical genetic model that recognised the disparity in distribution between unilateral and bilateral disease and that introduced the two-hit hypothesis [2]. Unilateral retinoblastoma is the most common form of the disease, representing 60% of cases. Most cases of unilateral retinoblastoma (85%) develop in the retina as a result of somatic mutations of the retinoblastoma gene. Previous studies have demonstrated that unilateral retinoblastoma is characterised by late presentation and hence a worse ocular outcome, eventually necessitating the enucleation of the affected eye. In cases of retinoblastoma, it is necessary to conduct genetic analysis in order to exclude hereditary germline disease and accordingly tailor counselling and follow-up plans for both eyes. This is of special importance in the very young (<12 months of age) with unilateral disease [3]. Lack of facilities for genetic testing in under-resourced countries imposes an added burden on clinical teams and families, unduly increasing the need for follow-up visits in order to closely monitor the unaffected eye. Our team has integrated an age-based algorithm to provide further follow-up sessions for patients presenting at <12 months of age. This algorithm favours the enucleation of unilateral-disease treatment-resistant C/D groups or D/E groups at presentation.

During the course of management of unilateral retinoblastoma, physicians typically prioritise overall survival above ocular survival. On the other hand, bilateral retinoblastoma imposes the extra burden of the need to salvage at least one eye. In bilaterally advanced disease, attempts to salvage one eye may compromise patients’ lives or act as a confounder for the statistical analysis. Also, setting an appropriate plan of management is often hindered by lack of studies that could have examined the broader aspects of childhood retinoblastoma or compared modalities of treatment. Most published studies have focused on the outcome of treatment of a single clinical or histological subgroup of patients or the use of a single management protocol.

In this study, we aimed at following a group of patients with unilateral retinoblastoma from presentation until their final follow-up stations. We concluded that such an analysis would provide a broader view of the management of retinoblastoma in limited-resource settings.

## Methods

### Data collection

Children’s Cancer Hospital Egypt (CCHE) has developed standard treatment plans for patients diagnosed with intraocular retinoblastoma, based on the latest available scientific evidence. Protocols were accepted by institutional scientific and ethics committees. Informed consent was obtained from parents or guardians before the start of treatment. The present study adhered to the guidelines of the Declaration of Helsinki and the International Conference of Harmonisation. All children were found too young to obtain assent. The hospital research database was queried for patients with intraocular retinoblastoma diagnosed between 7 July 2007 and 6 July 2015. The patients were followed up until 31 January 2016.

### Clinical aspects

**Fig. 1:**
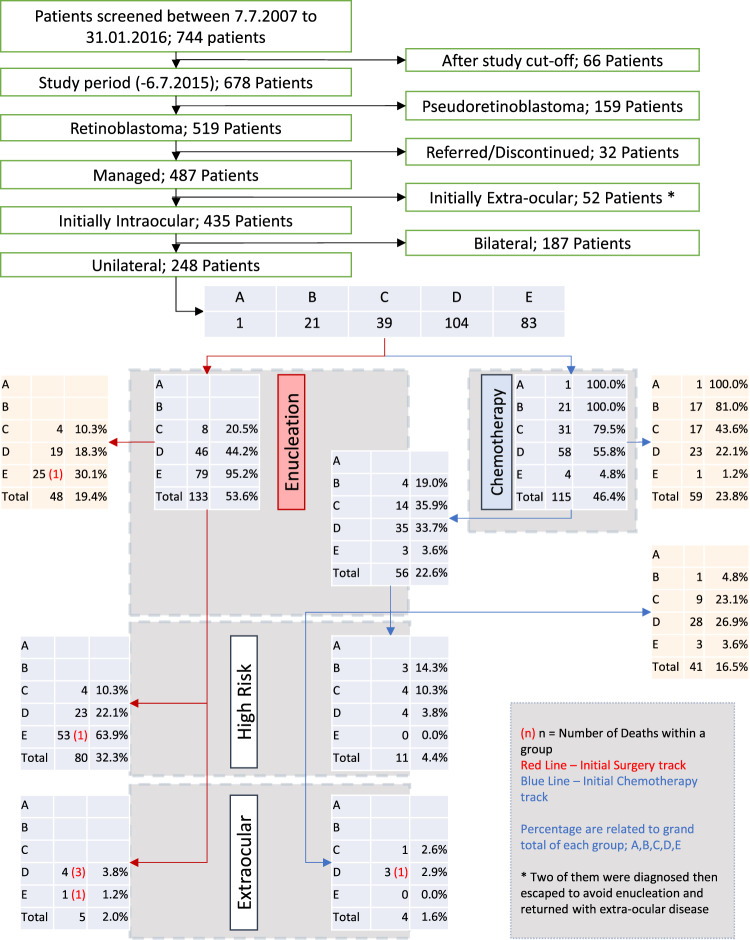
Selection and management of the patients in the study. Orange boxes: patients completed the treatment without highrisk features or extraocular disease.

Patients diagnosed with extraocular retinoblastoma 2 months after presentation at the hospital were considered as “initially presenting with extraocular disease” and were removed from the study. The International Intraocular Retinoblastoma Classification was used for ocular classification, while International Retinoblastoma Staging was used for extraocular disease [4]. The multi-disciplinary tumour board at CCHE discussed the management plans of all patients before the start of treatment and before any decision on enucleation, radiation or palliation. Figure 1 shows the algorithm used to select patients for this study. Paediatric oncologists and ophthalmologists examined patients at presentation. Examination was conducted under anaesthesia using RetCam II. Ultra-sonographies of the eye, as well as MRIs of the brain and orbit, were also conducted during the 1st week.

### Treatment

Patients were treated according to their disease classification at initial presentation; group A was managed using local laser and cryotherapy, while group B patients were treated using chemotherapy and local treatments. Groups C and D patients were treated according to patient age and predicted prognosis due to the absence of genetic testing at the hospital. Patients younger than 1 year of age were offered chemotherapy at the offset, while older patients underwent initial enucleation. Interim assessment was conducted after the second cycle of chemotherapy. For group E patients, enucleation was advised at diagnosis. One patient diagnosed as a group A patient was treated with laser therapy. The tumour, however, progressed and required systemic chemotherapy. Patients older than 1 year with favourable but incomplete response to chemotherapy were offered radiation therapy followed by re-evaluation. We excluded two patients whose parents/guardians had failed to participate in consultations with regard to the need for enucleation, and then returned later with extraocular disease. Our counselling sessions focused on the importance of enucleation in groups C and D, beginning with those diagnosed in 2011. Gross pathological examination was conducted on the same day of enucleation, and histopathological examination was done after fixation and staining by two senior pathologists according to the recommendations of the International Retinoblastoma Staging Working Group [5]. Guided by the definitions of the Children’s Oncology Group (COG), high-risk features were defined as massive invasions of the choroid or of the optic nerve beyond the lamina cribrosa or any degree of combined optic nerve and choroid invasion [6]. Vincristine–etoposide–carboplatin (VEC) were used as both neoadjuvant and adjuvant regimens of chemotherapy [7]. Resistance to chemotherapy, radiation and progression of the disease was an indication for enucleation. Patients with extraocular retinoblastoma were treated using the intensive multi-modality therapeutic plan of the COG (COG trial ARET0321) [8].

### Data analysis

We used the Research Electronic Data Capture database for data collection [9]. We used SPSS version 24 for data cleaning and analysis [10]. Survival analysis was conducted using the Kaplan–Meier method. Survival time was calculated from the time of diagnosis. Ocular survival was defined as the time between first presentation at the hospital and enucleation (or last follow-up date). The mean survival time represented the area under the survival curve in the interval 0 to *t*_max_ [11]. The Log-rank chi-square test was used to compare survival distributions, and *P* < 0.05 was considered significant.

## Results

### Demographical distribution

**Fig. 2:**
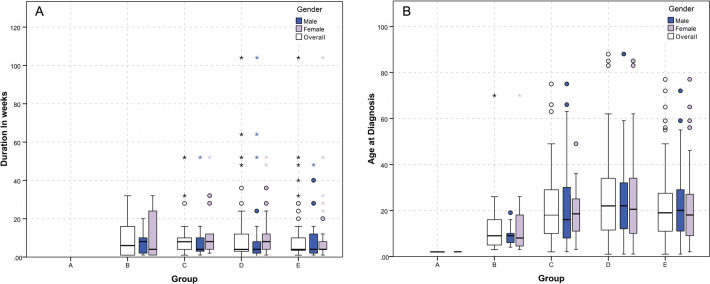
Comparison of age at diagnosis and duration of symptoms between groups and highlighted by gender. **A** Duration of symptoms in weeks. **B** Age at diagnosis in months.

During the study period, 248 patients were diagnosed with unilateral retinoblastoma, representing 56.49% of the patients that had presented with intraocular retinoblastoma (*n* = 439). Almost half of the sample were male (52.4%; *n* = 130), and 118 or 47.6% were female. The majority of the cases were Egyptian 96% (*n* = 238), while the rest came from Yemen (five), Sudan (three), Palestine (one) and Iraq (one). Mean age at diagnosis stood at 22.47 months (SD = 17.6, median = 18.5, range = 88). Patients referred from different regions of Egypt showed roughly similar distribution; 72 patients were referred from Nile Delta governorates, 87 from Greater Cairo and 67 from Upper Egyptian governorates (Table 1). Most patients were diagnosed with advanced intraocular disease (group D; *n* = 104, 41.9%, and group E; *n* = 83, 33.5%). Leukocoria was the main symptom; alone in 104 patients (41.9%) and combined with other symptoms in 10 patients (4%). Other symptoms included an abnormal look (*n* = 74, 29.8%) and strabismus (alone in 31 patients (12.5%) and combined with other symptoms in 11 patients (3.4%)). A number of patients also suffered from decreased visual acuity, lacrimation, redness and nystagmus. The mean duration of symptoms before diagnosis—as reported by parents/guardians—was 10.34 weeks (SD = 14.9, median = 4 weeks). There was no significant difference between genders with regard to age at diagnosis or duration of symptoms (Fig. 2). Only ten (4%) patients reported a family history of cancer; in five (2%) of these the family member had also reportedly suffered from retinoblastoma.

**Table 1:** Patients’ characteristics.

Variable	Description
Age at diagnosis	*n*; valid = 248
	Mean = 22.47, SD = 17.6
	Median = 18.5, range = 87.0
	Minimum = 1.0, maximum = 88.0
	First year of age: 87, 35.1%; second: 72, 29.0%; third and older: 89, 35.9%
Duration of symptoms (weeks)	*n*; valid = 227, missing = 21
	Mean = 10.34, SD = 14.93
	Median = 4.00, range = 104
	Minimum = 0, maximum = 104
Follow-up time (months)	*n*; valid = 248
	Mean = 32.85, SD = 22.85
	Median = 24.50, range = 92.00
	Minimum = 1.00, maximum = 92.00
Gender	Male 130, 52.4%
	Female 118, 47.6%
Patients’ distribution	Egypt: Delta and Northwest: 72, 29%
	Greater Cairo: 87, 35.1%
	South Nile Area and South West: 67, 27%
	Sinai, Suez Canal, and Red Sea: 12, 4.8%
	Non-Egyptians: 10, 4% (Yemen: 5, Sudan: 3, Palestine: 1, Iraq: 1)
Group of the eye	A, *n* = 1, 0.4%
	B, *n* = 21, 8.4%
	C, *n* = 39, 15.6%
	D, *n* = 104, 42.0%
	E*, n* = 83, 33.2%
Presentation	Leucocorea alone, *n* = 104, 41.9%
	Abnormal look, *n* = 74, 29.8%
	Squint, *n* = 31, 12.5%
	Decreased visual acuity, *n* = 6, 2.4%
	Leucocorea and squint, *n* = 9, 3.6%
	Lacrimation and redness, *n* = 3, 1.2%
	Decreased visual acuity and squint, *n* = 2, 0.8%
	Redness and blindness, *n* = 1, 0.4%
	Leucocorea and nystagmus, *n* = 1, 0.4%
	Not reported, *n* = 17, 6.8%

### Treatment

A total of 115 (46.3%) patients received neoadjuvant chemotherapy, and 133 (53.7%) underwent upfront enucleation. Fifty-six of those who had received neoadjuvant chemotherapy (22.5% of total patients; 48.7% of the neoadjuvant chemotherapy group) underwent enucleation after chemotherapy, and 15 patients required further chemotherapy after enucleation. Eighty-five patients (35% of total patients; 63.9% of the upfront surgery group) required further chemotherapy after an initial enucleation (Fig. 1). Seventeen (6.85%) of patients received external radiation therapy: 7 as a part of management of extraocular disease and 10 as a part of management of intraocular disease.

### Outcome

**Fig. 3:**
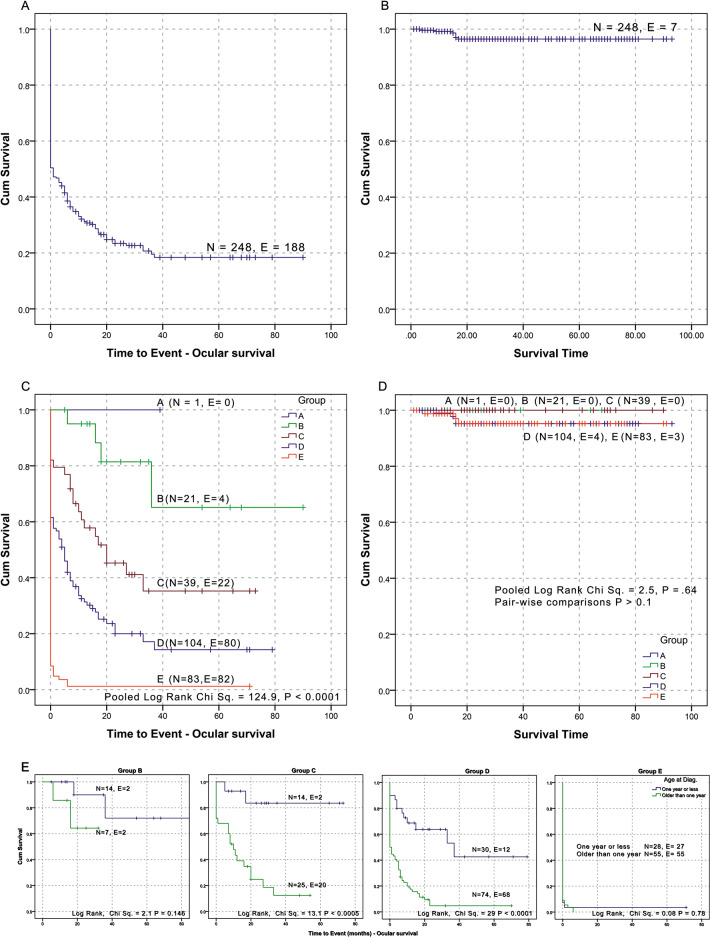
Ocular and overall survival of unilateral retinoblastoma. **A** Ocular survival. **B** Overall survival. **C** Ocular survival by group. **D** Overall survival by group. **E** Ocular survival in the different classification groups between patients diagnosed before one year of age and older than one year.

Mean ocular survival time was 20.76 months (95% CI: 16.03–25.49). An ocular survival plateau was reached at 37 months with a survival of 18.4%. There was a significant difference between each of the groups (*P* = 0.008 or less between each of B–E groups and other groups) and pooled over all groups (*P* < 0.00001). Group B was stable at 65.1% after 36 months; group C at 35.3% after 33 months; and group D at 14.3% after 37 months (Fig. 3). Figure 3E details the degree of success at salvaging eyes by age group.

Six out of seven patients who passed away had been lost to essential follow-up for periods in excess of 3 months, compared with 11 out of 241 surviving patients (chi-square *P* > 0.0001). The mean overall survival time was 90.19 months (95% CI: 88.14–92.24). There were no deaths in patients with intraocular group A, B, or C. There were seven deaths in the two other groups: four in group D and three in group E (3.74% of groups D and E combined; 2.82% of total patients) before a 17-month survival plateau of 96.4% was reached. No significant differences were found in overall survival rates between any two groups, or overall after pooling data from all groups. Nine (3.63%) patients developed extraocular disease at a later point of management: five after upfront enucleation and four patients after neoadjuvant chemotherapy (Fig. 1). Five patients had stage II (eye enucleated; microscopic residual tumour present). In addition, one developed stage IV metastatic disease (a-2) in the form of hematogenous metastasis with multiple lesions. Three other patients developed stage IV metastatic disease (b-3) with CNS extension and leptomeningeal disease.

## Discussion

Retinoblastoma is a tumour that afflicts children’s eyes and that results mostly from mutations of the retinoblastoma gene. Germline mutations put children under higher risk of developing bilateral disease, while somatic mutations are predictive of unilateral retinoblastoma. Therefore, unilateral retinoblastomas comprise all non-hereditary retinoblastomas and around 15% of familial and sporadic hereditary retinoblastomas [12]. Moreover, germline mutations may result in the very early development of retinoblastoma, which may sometimes be detectable during intrauterine life. Optimally, genetic testing would help identify patients with germline mutations and those with somatic mutations, and help predict the metachronous bilateral disease [13]. However, such testing is not available in developing countries due to the complexity of the test that requires specialised laboratories. Although older age does not exclude the presence of germline mutations [14], the relative contra-indication of radiation therapy for younger patients was the base of our decision to use the age limit of 1 year as a cut-off for preferring eye-salvage treatment over the enucleation for younger patients.

There is a consensus that group A retinoblastomas should be treated locally using laser for posterior tumours and cryotherapy for anterior tumours. Group B retinoblastomas are usually treated with chemotherapy plus local treatments. A study showed that treatment with minimised chemotherapy could give comparable results to standard VEC protocols [15]. There is a debate with regard to the proper management of groups C and D. In the case of equal bilateral disease, investigators have recommended trying chemotherapy, then assessing the response. In unequal disease between eyes, it has been recommended that the eye classified as “a group D eye” be enucleated if the tumour involves the macula and the other eye is a “group A or B eye” [16]. One study achieved the preservation of 82% of “group D eyes” using systemic chemotherapy alone in 47% of eyes, and almost doubled that success with using intensity-modulated radiation therapy.

In unilateral retinoblastoma, many studies have recommended enucleation as the first line of treatment for advanced disease. Others have recommended delaying enucleation of eyes of younger patients, citing the possibility of them developing metachronous bilateral disease [17, 18]. With “group E eyes”, there is a higher chance of finding high-risk pathological features post-enucleation. In these cases, chemotherapy could possibly mask extraocular spread. Therefore, many studies have conceded to the need for enucleating eyes at some stage of treatment in both unilateral and bilateral disease [17].

Other studies have shown that retinoblastoma could be managed using chemotherapy, even in advanced stages [19–21]. Before chemotherapy became a hallmark of treatment of retinoblastoma, ocular conservation studies in the late 1990s attempted to end the need for enucleation. Using radiation therapy, enucleation was avoided in 76–86% of eyes with advanced retinoblastoma, with ocular salvage rates starting at 41% after chemoreduction [22–24]. Our study showed similar patterns of success rates of chemoreduction as those reported by Shields et al. [21], but at lower percentages than their 100, 93, 90 and 47% success rates for groups A–D, respectively.

The debate intensified after the introduction of intra-arterial chemotherapy, where more significant concentrations of chemotherapy could be delivered selectively to the eye, allowing for better control of advanced intraocular tumours that were not manageable with traditional systemic intra-venous doses of chemotherapy. This approach has been used in developed countries even for unilateral advanced disease [25]. However, we would be cautious in widely using it in developing countries due to the hard follow-up and unavailability of standardised treatment strategies beside the current expensive methods involved that result in non-compliance of the patient for the multiple sessions.

As younger age carries an increased risk of developing retinoblastoma in the same and the other eye, and after considering the previously mentioned effects of radiation, we opted for recommending chemotherapy for young patients and enucleation for group D children older than 1 year of age [19, 26]. Previous studies have shown the importance of neoadjuvant chemotherapy in advanced unilateral retinoblastoma [27, 28]. Even in the presence of an intention to enucleate the eye, neoadjuvant chemotherapy could help eradicate any microscopic invasion of the optic nerve and orbital structures and facilitate the acquisition of postoperative free margins. We see our results to be in support of this practice. However, other studies have recommended against neoadjuvant chemotherapy [29]. It was suggested that chemotherapy would mask any distant metastasis. We believe that both regimens should be combined and that patients be counselled to the importance of compliance with a close follow-up schedule.

Even after complete regression or the other extreme of enucleation, close follow-up is mandatory because local recurrence can result from microscopically detectable remaining tumour cells. Close follow-up may also be essential for the early detection of systemic metastasis or other cancers that may develop after a germinal retinoblastoma (e.g., osteosarcomas, rhabdomyosarcomas or central nervous system tumours in trilateral retinoblastoma) [30, 31]. From our experience, enucleation of advanced eyes gave a false sense of security to some parents/guardians, resulting in low adherence to follow-up appointments (data are not shown).

The Canadian guidelines define “active follow-up” to be the period starting with the last active treatment and extending for 5 years or until the patient turns 9, after which the guidelines recommend the patient be put under long-term follow-up [3]. It would be interesting to investigate how much the discrepancy between the intended number of follow-up sessions and the actual number of sessions patients actually attend correlates with incidences of extraocular disease. In our study, six out of seven patients that had passed away were lost follow-up for periods longer than 3 months. Patients at risk of developing extraocular retinoblastoma were more likely to be lost to follow-up before getting diagnosed with extraocular disease. Patients with high-risk features did not display such behaviour. From our results, we believe that after initial enucleation, patients in groups D and E should be instructed to adhere to strict follow-up plans due to the risk of developing extraocular disease. Paradoxically, a larger portion of patients initially diagnosed as group D patients and treated with initial enucleation succumbed to disease (5/46) than group E patients (3/79). We believe that this resulted from incomplete follow-up, the presence of microscopic residual disease and group D parents/guardians having a false sense of security after the end of chemotherapy.

Our study could be more comprehensive if the data had been pooled from multiple centres in several developing countries using a standardised data collection scheme. More analysis could have been conducted on the extension of follow-up periods, correlation with socio-demographic and economic indicators in patients’ regions of origin. Two recent studies from the Global Retinoblastoma Study Group and an Indian group involved two of the largest groups of patients with retinoblastoma. They were both published during the preparation of this manuscript [32, 33]. The overall survival of unilateral retinoblastoma patients in the Indian study (90%) was lower than that in our study (96.5%), possibly because of the inclusion of patients with extraocular disease at presentation. We acknowledge such efforts and we call for the development of a standard protocol for the reporting of retinoblastoma that would allow for better meta-analyses. Such protocol should ideally include the reporting of detailed management plans and outcomes of unilateral and bilateral retinoblastoma separately.

In summary, we believe that our study should pave a road for the creation of a new benchmark for the management of unilateral retinoblastoma in developing countries where essential tools are available. We encourage centres to collaborate in coordinating resources, becoming a “single system” and providing healthcare services to patients at their nearest accessible point. Enucleation in unilateral disease should not represent the end of the relationship between patients with retinoblastoma and their managing physician.

## Summary

### What was known before

- Initial classification and close follow-up are important determinants of success of management of retinoblastoma.

### What this study adds

- Although initial chemotherapy is reserved for conservative management, adding initial chemotherapy reduces mortality even if enucleation is planned.
